# High content of low molecular weight organics does not always affect pharmaceutical adsorption on activated carbon: The case of acetate, propionate and ethanol in source-separated urine

**DOI:** 10.1016/j.wroa.2023.100199

**Published:** 2023-09-07

**Authors:** Aurea Heusser, Anne Dax, Christa S. McArdell, Kai M. Udert

**Affiliations:** a Eawag, Swiss Federal Institute of Aquatic Science and Technology, 8600 Dübendorf, Switzerland; b ETH Zürich, Institute of Environmental Engineering, 8093 Zürich, Switzerland

**Keywords:** Carbon usage rate, Pharmaceutical removal, Micropollutants, Competing adsorption

## Abstract

Adsorption on activated carbon is a common process to remove pharmaceuticals in wastewater treatment. Activated carbon adsorption is usually applied to wastewater with a low content of biological degradable organics, i.e. after biological treatment. Especially low molecular weight (LMW) compounds are known to compete with pharmaceuticals for adsorption sites. The goal of this study was to test the hypothesis that biological treatment is necessary for efficient pharmaceutical removal. Source-separated urine after anaerobic storage (anaerobically stored urine) and after aerobic biological removal of organics without nitrification (organics-depleted urine) were used in this study. In anaerobically stored urine 60% of the organic compounds were LMW organics, of which about 40% were acetate and propionate. 74% of the DOC and 100% of acetate and propionate were removed during aerobic biological treatment. To investigate the effect of the organic compounds on pharmaceutical removal, sorption experiments with 19 spiked pharmaceuticals and one artificial sweetener were conducted with powdered activated carbon. Ethanol, another LMW organic, was included in the study, as it is regularly used for pharmaceutical spiking thereby strongly increasing the DOC content. The experiments showed that the adsorption of the pharmaceuticals and the sweetener were hardly affected by the easily biodegradable LMW organics or ethanol. Therefore, it was concluded that biological pre-treatment is not necessary for efficient pharmaceutical adsorption. Since acetate, propionate and ethanol contribute substantially to the DOC content but do not absorb UV light, the latter is recommended as indicator for pharmaceutical removal in solutions with high contents of biodegradable LMW organics.

## Highlights

• Efficient removal of pharmaceuticals from untreated urine with activated carbon.
• Biodegradable low molecular weight organics do not hinder pharmaceutical adsorption.
• Even high concentrations of the LMW ethanol do not hinder pharmaceutical adsorption.
• UV absorption is a more generally applicable indicator of adsorption than DOC.

## Graphical abstract

**Figure:**
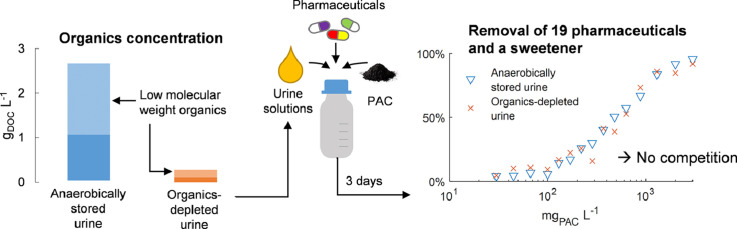

## Introduction

Pharmaceuticals in the environment pose a growing concern as they are persistent and especially the toxicity of mixtures is greatly unknown (Kümmerer, 2008). Most pharmaceuticals are excreted by humans and enter the environment via the wastewater path (Daughton and Ternes, 1999). For efficient removal of pharmaceuticals, conventional wastewater treatment is not sufficient, but an additional treatment step is necessary (Falås et al., 2016; Joss et al., 2005). A common treatment technology to remove pharmaceuticals from wastewater is adsorption to activated carbon in powdered or granular form (Metcalf et al., 2014). Activated carbon is an effective but unselective adsorbent for organic substances. The type of activated carbon influences the adsorption as shown by literature (Worch, 2021), but this influence was not the focus of the present study. In wastewater treatment, the presence of bulk organics reduces the adsorption capacity of pharmaceuticals on activated carbon by competing for adsorption sites (Matsui et al., 2003). Different properties of organics can drive competitiveness such as the existence of unsaturated structures, hydrophobicity and aromaticity (Wang et al., 2021) and the presence of specific functional groups and electrostatic interactions for ionic compounds (Kah et al., 2017). Size fractioning, for example with size exclusion chromatography, can identify the main competing fraction of organics. In several studies, the low molecular weight (LMW) organic fraction, with molecular weights smaller than 350 g mol^−1^ (Jacquin et al., 2017), was identified as the main competing fraction of the dissolved organic carbon (DOC), e.g. in drinking water (Newcombe et al., 2002), lake water (Velten et al., 2011) and wastewater (Zietzschmann et al., 2014). Mostly, LMW organics and neutrals, such as alcohols, aldehydes, ketones and sugars (Huber et al., 2011) were not differentiated. In wastewater treatment plants (WWTPs), pharmaceutical adsorption is typically done after the biological degradation of the organics, either with powdered activated carbon (PAC) (Boehler et al., 2012) or with granular activated carbon (GAC) filters (Benstoem et al., 2017). LMW organics are mostly easily biodegradable organics (Huber et al., 2011), so that the LMW content of the wastewater is low in the adsorption step.

Adsorption on activated carbon is also used for the removal of pharmaceuticals from source-separated urine. Separate collection of urine to recover nutrients can be an important contribution to a circular economy (Larsen et al., 2013) . In addition to the majority of nutrients, also about 64% of the active ingredients of pharmaceuticals are excreted via urine (Lienert et al., 2007). Separate collection and treatment of urine is therefore also a promising strategy for preventing environmental pollution with pharmaceuticals. However, the concentration of organics in urine is very high (Udert et al., 2006), Özel Duygan et al. (2021) reported DOC values between 1500 and 1800 mgC L^−1^ for anaerobically stored urine and it was expected that the high DOC content would result in a high demand for PAC for pharmaceutical removal. The organics in fresh urine were well characterized by Putnam (1971) and more recently by Bouatra et al. (2013) and comprise a list of more than 2500 substances. In most current separation systems, urine is stored anaerobically after collection, and the organics are fermented to LMW organics (Jacquin et al., 2018), predominantly acetate and propionate (Udert et al., 2013). However, a detailed characterization of anaerobically stored urine as for fresh urine is not available yet. In one of the most common urine treatment processes based on nitrification and distillation (Fumasoli et al., 2016), pharmaceuticals are removed in a GAC column after the removal of easily biodegradable organic compounds and nitrification (Köpping et al., 2020). While biological urine treatment usually includes organics degradation and nitrification, recent studies have shown that organics degradation in urine can easily be achieved without nitrification (Heusser et al., 2023). The product is an organics-depleted urine. The separation of organics degradation and nitrification could help to stabilize nitrification (Heusser et al., 2023). In general, biological urine treatment and municipal wastewater treatment have a very similar treatment train: the easily biologically degradable compounds such as LMW organics are biologically degraded, and concomitantly or subsequently, pharmaceuticals are removed by adsorption. However, the organics comprise different compounds. Raw wastewater is dominated by fibers, proteins and sugars (Huang et al., 2010) and in secondary wastewater effluent the organics comprise mainly humics and LMW acids (Zietzschmann et al., 2016) whereas fresh human urine is dominated by amino acids and derivatives, carbohydrates and carbohydrate conjugates (Bouatra et al., 2013) and anaerobically stored urine comprises mainly LMW acids (Udert et al., 2013). During nitrification of urine most LMW organics are degraded and lead to the production of biopolymers (Jacquin et al., 2018). Also the monitoring of pharmaceutical removal in urine treatment is done in a similar way as in municipal wastewater treatment. Indicators such as DOC (Altmann et al., 2014) and UV absorption (Wittmer et al., 2015) are used as surrogates for pharmaceuticals, as pharmaceutical measurements are cost-, time- and labor-intensive.

As outlined above, pharmaceutical removal by sorption to activated carbon in urine and wastewater treatment is usually done after most LMW organics were degraded. Furthermore, LMW organics have been identified as the DOC fraction, which competes the most with pharmaceuticals for adsorption on activated carbon. It has been generally presumed that pharmaceutical removal from urine with a high content of easily biodegradable LMW organics is not possible. However, to our knowledge this hypothesis has not been tested so far. This study addresses this knowledge gap, using anaerobically stored urine as an example as it contains most pharmaceuticals and has a high DOC concentration, mainly biodegradable LMW organics. To test the importance of the LMW organics, pharmaceutical removal by adsorption on activated carbon was compared for anaerobically stored urine and organics-depleted urine.

Ethanol was included in this study, because it is an important LMW organic in the neutral fraction (Ruhl and Jekel, 2012) often used in stock solutions when pharmaceuticals are spiked.

To summarize, this study was guided by the following two objectives:

1 Determine whether the adsorption of pharmaceuticals on activated carbon is hindered by high content of LMW organics in urine
2 Determine whether the LMW organic ethanol competes with pharmaceuticals for adsorption sites

## Results and discussion

### Low molecular weight (LMW) organics in anaerobically stored urine and organics-depleted urine

**Fig. 1:**
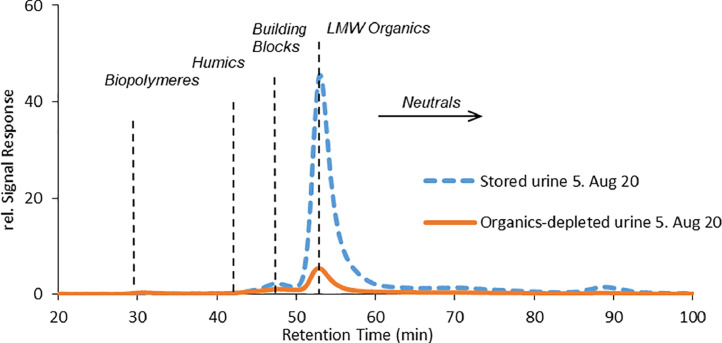
Size exclusion chromatogram of the organic carbon detector for anaerobically stored urine and organics-depleted urine (collected in August 2020).

All the urine solutions were sourced from the urine collection system at Eawag. The urine collected in May 2022 from the anaerobic storage tank had a DOC and dissolved chemical oxygen demand (COD) content of 676 mgC·L^−1^ and 1530 mgCOD·L^−1^, respectively (Table 1). Size exclusion chromatography (SEC) measurements in August 2020 showed that 66% of the DOC were LMW organics (Fig. 1, for more dates see Fig. S1 in the supporting information (SI)). The bases of volatile fatty acids, especially acetate and propionate, made up for 37% of the DOC or 62% of the LMW organics and were therefore the largest fraction of LMW organics. Their chromatograms are shown in Fig. 4 in section 2.4. The measurement included also butyrate, isovalerate, and valerate, which were all below the limit of quantification (LOQ) of 2 ppm and isobutyrate, which overlapped with carbonate and could therefore not be quantified (Table 1). In a previous study (Udert et al., 2013), acetate, propionate and butyrate were identified as main VFA bases with COD fractions of 47%, 4% and 6%, respectively. In our study, acetate was also the main VFA base with a slightly lower COD fraction of 38%, while propionate had a COD fraction of 6%.

**Table 1:** Characterization of the organics in anaerobically stored and organics-depleted urine (collected in May 2022). Isobutyrate could not be determined with ion chromatography due to an overlap with carbonate. The concentrations of butyrate, isovalerate, and valerate were below the limit of quantification (LOQ) of 2 ppm.

		Anaerobically stored urine	Organics-depleted urine	Removed in organics degradation
Acetate	ppm	542	<LOQ	100%
	mgC L^−1^	217	<LOQ	100%
	mgCOD L^−1^	578	<LOQ	100%
Propionate	ppm	64	<LOQ	100%
	mgC L^−1^	31	<LOQ	100%
	mgCOD L^−1^	97	<LOQ	100%
isobutyrate	ppm	n.a.	n.a.	n.a.
butyrate, isovalerate, valerate	ppm	<LOQ	<LOQ	n.a.
Sum VFAs	mgC L^−1^	248	0	100%
	mgCOD L^−1^	675	0	100%
DOC	mgC L^−1^	676	173	74%
CODsol	mgCOD L^−1^	1530	494	68%
VFA fraction	% of DOC	37%	0%	
	% of COD	44%	0%	
pH	–	9	8.8

Most of the organics, i.e. 74% of the DOC, were degraded in a membrane-aerated biofilm reactor (MABR) including all acetate and propionate but without nitrifying the ammonia, producing organics-depleted urine (Table 1). SEC measurements in August 2020 revealed that 85% of the LMW organics were removed, which means that LMW organics accounted for 76% of the DOC removal. Nearly half of the degraded compounds were acetate (43% of the degraded DOC) and propionate (6% of the degraded DOC). In organics-depleted urine the LMW organics and neutrals together account for 53% of the organics. The share of all the other fractions (biopolymers, humics, building blocks) increased compared to anaerobically stored urine but only the concentration of biopolymers increased by about 33% compared to anaerobically stored urine. During biological treatment the pH decreased only slightly from 9.0 to 8.8, and also the nutrient concentrations were hardly affected (Table 2). Due to the high pH of 9 in anaerobically stored urine, nitrification was inhibited (Fumasoli, 2016).

**Table 2:** Urine characterization for the different urine solutions used in the experiments before spiking of pharmaceuticals. Nitrate and nitrite were not detected in any urine solution.

**Experiment**	**Urine collection date**	**Urine solution**	**DOC**	**COD_sol_**	**pH**	**NH_4_^+^**	**PO_4_^3−^**	**Cl^−^**	**Na^+^**	**K^+^**
			mg L^−1^	mg L^−1^	–	mgN L^−1^	mgP L^−1^	mg L^−1^	mg L^−1^	mg L^−1^
Normal operation	05.Aug 20	Anaerobically stored urine	1280	n.a.	n.a.	3050	211	2150	1250	820
		Organics-depleted urine	311	n.a.	n.a.	2820	235	2360	1350	856
Organics measurement	23.May 22	Anaerobically stored urine	676	1530	9	2120	111	1750	1020	895
		Organics-depleted urine	173	494	8.8	1770	109	1790	1010	928
		Removed in organics degradation	74%	68%		16%	2%	−2%	1%	−4%
PAC experiment	25.Nov 21	Anaerobically stored urine	2700	n.a.	n.a.	2500	130	2300	1200	1000
		Organics-depleted urine	300	762	n.a.	3400	220	2400	1400	1000
PAC experiment with UV measurement	30.Jul 21	Anaerobically stored urine	1340	n.a.	8.6	3460	200	2460	1480	1030
	26.Mar 21	Organics-depleted urine	190	n.a.	8.8	2740	232	2140	1150	750

n.a. = not available.

### The effect of biodegradable LMW organics on the pharmaceutical removal by PAC

**Fig. 2:**
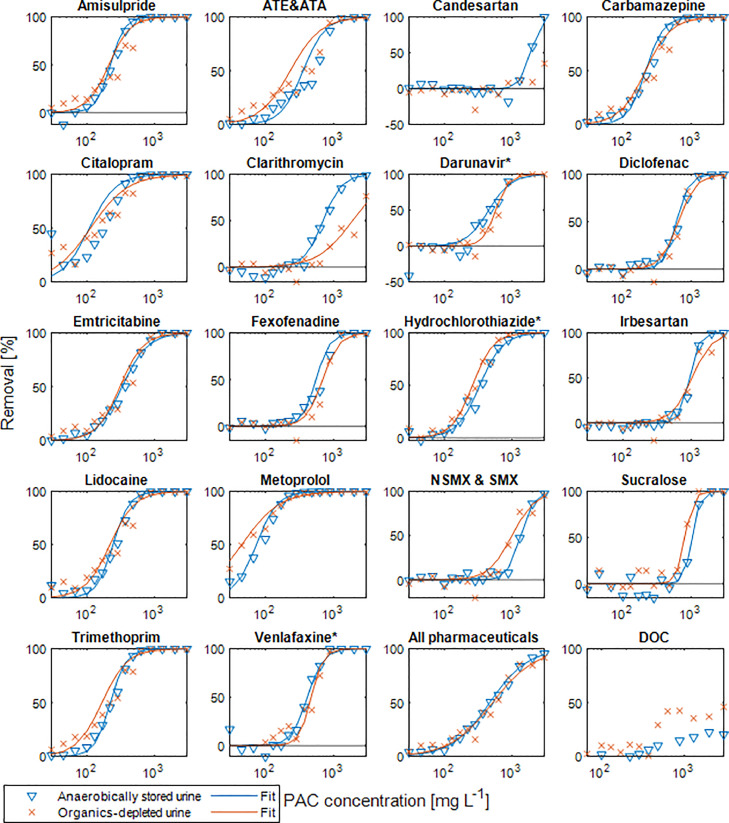
Removal of pharmaceuticals by activated carbon in anaerobically stored and organics-depleted urine (see Table 2, urine collected in November 2021) after three days and fitted with Eq. (2). Removal of less than 10% was excluded for the fitting. ATE & ATA is the sum of atenolol and atenolol acid and NSMX &SMX is the sum of sulfamethoxazole and N_4_-acetylsulfamethoxazole and "All pharmaceuticals" includes also the sweetener sucralose. For substances highlighted with an asterisk (*), the concentrations in the batch without PAC (the reference) was an outlier, the concentration in the batch with 45 mg PAC L^−^^1^ was used as a reference instead (see Fig. S5 in S4 for details).

The effect of easily biodegradable LMW organics on pharmaceutical removal was tested by dosing different PAC concentrations to anaerobically stored urine and organics-depleted urine, collected in November 2021 and spiked with one artificial sweetener, sucralose (SUC), and 19 pharmaceuticals, amisulpride (AMS), atenolol and atenolol acid (ATE & ATA), candesartan (CAN), carbamazepine (CAR), citalopram (CIT), clarithromycin (CLA), darunavir (DAR), diclofenac (DIC), emtricitabine (EMT), fexofenadine (FEX), hydrochlorothiazide (HCT), irbesartan (IRB), lidocaine (LID), metoprolol (MET), N_4_-acetylsulfamethoxazole and sulfamethoxazole (NSMX & SMX), trimethoprim (TMP), and venlafaxine (VEN). The target concentration for all pharmaceuticals and the artificial sweetener were 200 µg L^−1^, but the final concentrations varied. Strong deviations were due to presence of some of the pharmaceuticals in the urine solutions (see Table 4 in the Materials and methods section). Despite the difference of the initial DOC concentration in anaerobically stored urine with the exceptionally high DOC of 2670 mgC L^−1^ and organics-depleted urine with 280 mgC L^−1^, the removal of most pharmaceuticals and of the artificial sweetener SUC did not differ significantly between the two urine solutions (Fig. 2), which means that the biodegradable LMW organics did not substantially affect pharmaceutical removal by activated carbon adsorption. The curve fitting, using the simplified equivalent background compound model (SEBCM) including the 95% confidence intervals, outlier detection and correction is shown in section S4 in the SI. Fig. 2 reveals a slightly better removal from anaerobically stored urine compared to organics-depleted urine for CAN and CLA. However, for these substances only four and two data points, respectively, were available as these substances were not sorbing very well, making the analysis uncertain (see Fig. S7 and Table S1 in S4 for more details). The pharmaceutical concentrations were hardly affected when no PAC was added, more than 20% removal was observed only for CIT, HCT and IRB over the three days (see Fig. S4 in S3 for more details).

### The effect of ethanol on the pharmaceutical removal by PAC

**Fig. 3:**
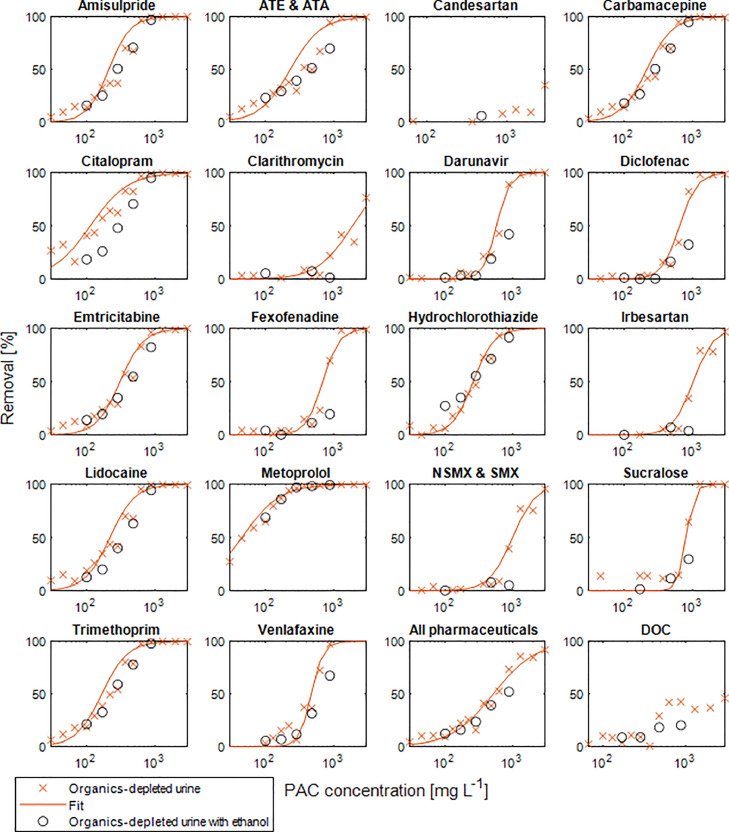
Effect of additional ethanol (204 mgC L^−^^1^) on the removal of pharmaceuticals by activated carbon from organics-depleted urine (see Table 2, urine collected in November 2021) after three days and fitted with Eq. (2). Removal less than 10% was excluded for the fitting. For the organics-depleted urine with additional ethanol not enough data was available for fitting. ATE & ATA is the sum of atenolol and atenolol acid and NSMX &SMX is the sum of sulfamethoxazole and N_4_-acetylsulfamethoxazole and "All pharmaceuticals" includes also the sweetener sucralose.

Ethanol is usually not present in anaerobically stored or organics-depleted urine but the spiking of pharmaceuticals with ethanol as solvent increased the DOC concentration in anaerobically stored urine (2670 mgC L^−1^) by 130 mgC L^−1^ to 2800 mgC L^−1^, corresponding to a 5% increase and in organics-depleted urine (284 mgC L^−1^) by 202 mgC L^−1^ to 486 mgC L^−1^, corresponding to a 70% increase. To determine the effect of ethanol on the adsorption of pharmaceuticals, batch experiments were performed using organics-depleted urine spiked with pharmaceuticals with and without a further addition of ethanol by 204 mgC L^−1^ to 690 mgC L^−1^ DOC. The removal of the individual pharmaceuticals shown in Fig. 3 indicate that ethanol does not substantially influence the adsorption of the pharmaceuticals. Not enough data for a curve fitting with ethanol addition were available. The curve fit of the organics-depleted urine with the 95% confidence interval together with the data points for ethanol addition are shown in Fig. S8 in S4. The deviation for CIT removal is probably due to a measurement error in the reference sample without PAC dosage as discussed in section S4 in the SI. The DOC removal has a high variability and no clear difference for different ethanol addition could be observed.

These results confirm findings from literature. Wang et al. (2021) showed that ethanol is not a strong competitor for adsorption of pharmaceuticals since alcohols with shorter chain lengths show lower adsorbability to PAC, however the concentration used was much lower with 10 mgC L^−1^. Furthermore, Özel Duygan et al. (2021) showed that reducing the ethanol used as solvent for the pharmaceutical by a factor of 10 (i.e. for DOC increase by the addition of ethanol of factor 14 and 2.5, respectively) did not change the result of adsorption to PAC for pharmaceuticals in nitrified urine.

### Adsorption of LMW organics on PAC

**Fig. 4:**
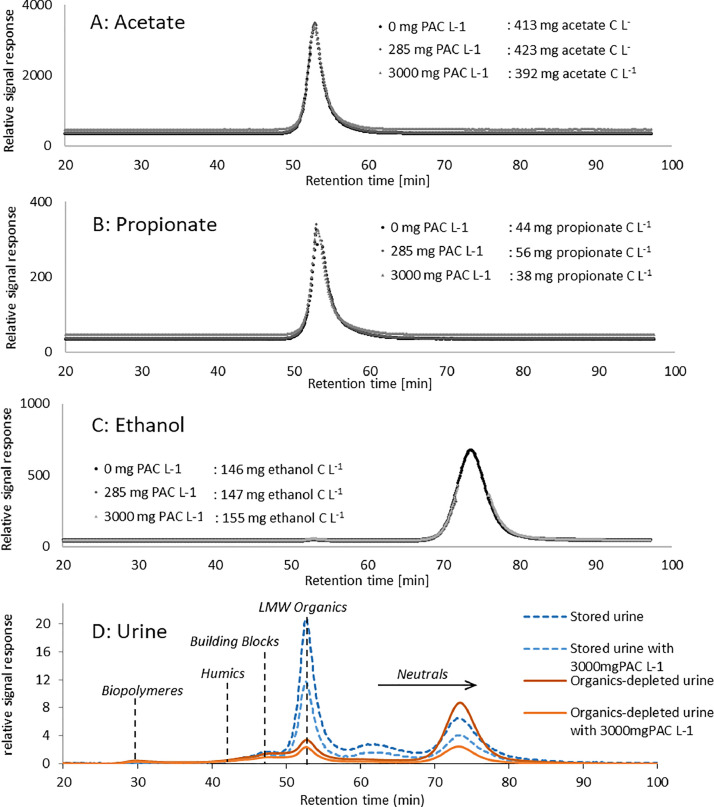
Size exclusion chromatogram for [A] acetate, [B] propionate, [C] ethanol and for [D] anaerobically stored urine and organics-depleted urine (collected in November 2021), spiked with pharmaceuticals in ethanol (peak at 73 min) and treated with different concentrations of powdered activated carbon (PAC). The different size fractions are indicated in [D].

The results discussed above suggest that easily biodegradable LMW organics, especially acetate and propionate and ethanol, do not adsorb on activated carbon because they did not hinder pharmaceutical adsorption. To test this hypothesis, PAC adsorption experiments were conducted with nano-pure water and acetate, propionate and ethanol respectively. In the SEC measurements acetate and propionate showed up in the fraction of LMW organics, both having the maximum of their peak at 53 minutes retention time and ethanol has a longer retention time of 73 minutes falling in the fraction of neutrals, shown in Fig. 4. The SEC measurements with PAC show that in none of the samples, even at the very high PAC concentration of 3000 mg L^−1^, the concentration of acetate, propionate, or ethanol changed substantially (Fig. 4). These results confirm the assumption that adsorption of acetate, propionate and ethanol on activated carbon is negligible and that this is the reason, why they do not hinder pharmaceutical adsorption.

While acetate, propionate and ethanol did not adsorb, other LMW organics were the constituents of DOC which adsorbed the most. The addition of PAC to anaerobically stored and organics-depleted urine mainly removed LMW organics as shown in Figs. 4 and S2 in S1. Nutrients were hardly affected by the addition of PAC as shown in Fig. S3 in S2. The peak in the neutral fraction (73 min) can be attributed to ethanol because it is not present in non-spiked urine solutions as shown in Fig. 1.

High adsorption of LMW organics was reported in literature before, but with solutions where the LMW organics were not biodegradable, e.g. WWTP effluent or natural waters. For example Zietzschmann et al. (2014) found fast adsorption of LMW organics to PAC from WWTP effluent and Altmann et al. (2015) showed that biopolymers in WWTP effluent adsorb less compared to organics with a lower molecular weight. Also Velten et al. (2011) showed for different GAC that the absorbability increases with decreasing molecular size for lake water. We were able to show that the presence of LMW organics in urine does not hamper adsorption of pharmaceuticals. Hence, a high concentration of LMW organics does not necessarily mean that adsorption of pharmaceuticals is unfeasible. Consequently, the competition with pharmaceutical adsorption depends on the individual compounds.

### Indicators for pharmaceutical removal

**Fig. 5:**
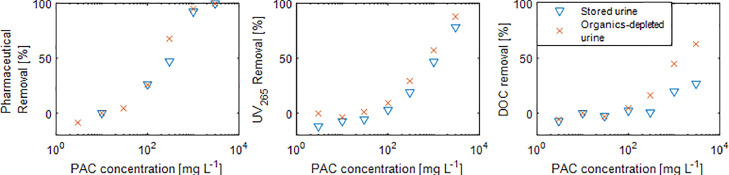
Average removal of 19 pharmaceuticals and an artificial sweetener, UV_265_ absorbance removal and DOC removal at different PAC concentrations for anaerobically stored and organics-depleted urine (collected in July and March 2021).

UV absorbance and DOC removal are used in wastewater and urine treatment as indicators for pharmaceutical removal with activated carbon. The correlation of UV absorbance reduction and pharmaceutical removal is based on the observation that UV-absorbing organics are readily adsorbed on activated carbon (Shimabuku et al., 2017). In wastewater treatment, 254 nm is commonly used for UV analysis (Altmann et al., 2016), but a wavelength of 265 nm is recommended if nitrate concentrations are high to prevent interference (Köpping et al., 2020). Measurements of acetate, propionate, ethanol and the pharmaceutical mix, each dissolved in nano-pure water, demonstrated that only pharmaceuticals but neither acetate, propionate nor ethanol absorb UV light substantially at 265 nm and 254 nm (see Fig. S9 in S5). However, acetate, propionate and ethanol increase the DOC concentration, suggesting that UV absorbance is a more reliable indicator for pharmaceutical removal in the presence of easily biodegradable LMW organics. In a second set of PAC experiments with anaerobically stored and organics-depleted urine collected in July 2021 and March 2021 with a DOC after spiking of 1340 mgC L^−1^ and 190 mgC L^−1^, respectively, the pharmaceutical removal was similar for both urine solutions (Fig. 5). The results for each substance are shown in section S5. The same was observed for the first set of PAC experiment (Fig. 2). The data also show that the UV_265_ removal was similar for both urine solutions as well and ranged from 0% to 88% (Fig. 5). However, the DOC removal for both solutions was different, which can be explained by the difference of constituents of DOC that do not adsorb. The deviation of the DOC removal for the different urine solutions was also observed for the first set of PAC experiments (Fig. 2, last subplot). The DOC removal in Fig. 5 was 27% in anaerobically stored urine and 63% in organics-depleted urine at a PAC dose of 3000 mg L^−1^, so exhibiting a lower resolution than the UV removal. The lower resolution of DOC removal, the significant variability, and the apparent dependency on the initial DOC concentration renders DOC removal an unsuitable indicator for pharmaceutical removal in urine. Utilizing UV_265_ removal as an indicator can help alleviate these limitations. The correlation of pharmaceutical removal and UV_265_ removal is shown for the individual substances in Fig. S10 in S5.

## Conclusions

• Adsorption of pharmaceuticals from anaerobically stored urine is not hampered by the high concentration of easily biodegradable LMW organics.
• The most abundant organic substances in anaerobically stored urine are acetate and propionate; these LMW organics do not adsorb on activated carbon and therefore they do not compete for adsorption sites with pharmaceuticals.
• Ethanol, used for spiking pharmaceuticals, does not adsorb on activated carbon and therefore it does not compete for adsorption sites with pharmaceuticals.
• The reduction of UV-absorbance at 254 nm and 265 nm as an indicator for pharmaceutical removal is independent of the background organic matter. In contrary, the correlation of DOC and pharmaceutical removal is more variable and is different in anaerobically stored urine and organics-depleted urine.
• Our study shows that a high concentration of LMW organics does not necessarily mean that the competition for adsorption sites is high. The actual composition of the LMW organics is decisive for competition with pharmaceuticals adsorption. As a consequence, the usage of activated carbon for pharmaceuticals removal could be considered for some liquid waste streams with high LMW content.

## Materials and methods

### Urine treatment and characterization

Urine was collected from the urine treatment system in the NEST building on the Eawag/Empa campus in Dübendorf. The system is equipped with waterless urinals and urine-diverting toilets (Save!, Laufen AG), which allow the separation of urine with approximately 200 mL flushing water entering the urine pipe per flush. The dilution with flushing water is around 1:1, calculated based on the salt concentration presented in Table 2 and in undiluted stored urine presented in Udert et al. (2006). This dilution is higher than normal because we had only few users during urine collection while the cleaning frequency remained the same. The urine was stored anaerobically in a tank in the basement with a variable hydraulic retention time of 2 to 8 weeks depending on the production of urine and the performance of the treatment. The urine taken from the storage tank is referred to as anaerobically stored urine. Biological degradation of organics was performed in a 60 L membrane aerated biofilm reactor (MABR) (Oxypilot, Oxymem) with a hydraulic retention time (HRT) of about 1.2 days. The pH of the anaerobically stored urine was 9 (Table 2), which inhibits nitrification (Fumasoli, 2016). The MABR was operated with continuous inflow of approximately 2 L h^−1^ and continuous aeration with pressurized air at a flow rate of 200 L h^−1^. The pressure was kept at 300 mbar, scouring was performed weekly and sludge removal of 2 L was performed monthly. The treatment in the MABR removed between 70% and 90% of the organics present in anaerobically stored urine, and after treatment it was referred to as organics-depleted urine. The concentration of the organics in the anaerobically stored urine varied over time due to changing user behavior, e.g. higher dilution during less usages while keeping the cleaning frequency. The characterization of the urine solutions used for the experiments is given in Table 2.

### Experimental setup

For sorption experiments with PAC, the granular activated carbon Norit® GCN 830 (properties in Table S4 in S8) was used after washing it three times with nano-pure water and drying it at 105 °C. The GAC was milled to a mean diameter of 16 μm in a ball mill (MM400, material: inox, Retsch®) at 30 Hz twice for two minutes. The PAC was once again dried over two days in an oven at 105 °C and cooled one day in a desiccator before producing a 10 mg L^−1^ PAC suspension with nano-pure water. The PAC solution was stirred at 150 rpm before and during pipetting the solution in the batches.

The anaerobically stored and organics-depleted urine was filtered using a 0.45 μm MN GF-5 filter (Macherey-Nagel) for all batch experiments. The collected urine was spiked with the micropollutant mix to achieve 200 μg L^−1^ of each substance (see section 3.3), resulting in a DOC increase of 150 and 200 mgC L^−1^ for stored and organics-depleted urine due to the ethanol addition. The spiked urine collected in November 2021 was filled in 250 mL Schott bottles and PAC solution was added to achieve logarithmically distributed PAC concentrations of 0, 30, 45, 67, 100, 130, 170, 220, 285, 370, 480, 630, 880, 1300, 2000, 3000 mg L^−1^ as indicated in Table 3. This range covered the pharmaceutical removal by 0 to 100% for most substances from the urine solutions. For the organics-depleted urine collected in November 2021 another six batches were prepared with an additional DOC increase by ethanol of 204 mgC L^−1^, with 0, 100, 170, 285, 470, and 880 mgPAC L^−1^. A second set of PAC experiments with organics-depleted urine and stored urine collected in July and March 2021, respectively, was performed with PAC concentrations of 0, 3, 10, 30, 100, 300, 1000, 3000 mg L^−1^ and for this second set of PAC experiments UV absorption measurements were included. The batches were mixed in an overhead shaker for three days to make sure the equilibrium concentration was achieved, as 24 hours are advised for PAC batch experiments to achieve equilibrium (Böhler, 2019).

**Table 3:** Overview of the conducted experiments.

**Experiment (goal)**	**Urine collection date**	**Solution**	**DOC Conc. (after spiking)**	**Spiking conc.**	**PAC conc.**	**Duration**
			mg L^−1^	µg L^−1^	mg L^−1^	days
Normal operation (organics characterization)	05. Aug 20	Anaerobically stored urine	1340	–	–	–
		Organics-depleted urine	190	–	–	–
PAC experiment (adsorption of pharmaceuticals with competing LMW organics)	25. Nov 21	Anaerobically stored urine	2800	200	0, 30, 45, 67, 100, 130, 170, 220, 285, 370, 480, 630, 880, 1300, 2000, 3000	3
	25. Nov 21	Organics-depleted urine	486	200		3
PAC experiment (adsorption of pharmaceuticals with ethanol)	25. Nov 21	Organics-depleted urine with additional ethanol	690	200	0, 100, 170, 285, 470, 880	3
PAC experiment (adsorption of acetate, propionate and ethanol)		Acetate	400	–	0, 285, 3000	1
		Propionate	50	–		1
		Ethanol	150	–		1
PAC experiment with UV measurement (adsorption of pharmaceuticals with competing LMW organics and UV absorption)	30. Jul 21	Anaerobically stored urine	1340	200	0, 3, 10, 30, 100, 300, 1000, 3000	3
	26. Mar 21	Organics-depleted urine	190	200		3

Acetate, propionate and ethanol were diluted in nanopure water to 400 mgC L^−1^, 50 mgC L^−1^ and 150 mgC L^−1^, respectively, as indicated in Table 3. PAC was added at concentrations of 0 mg L^−1^, 285 mg L^−1^, and 3000 mg L^−1^ to the three solutions and they were shaken during 24 h on a plate shaker (Table 3).

Samples were taken before and at the end of the experiment, 5 mL was stored separately and frozen at −20 °C immediately for later analysis of the pharmaceuticals. General parameters (see section 3.4) were measured right after sampling.

### Selection of the pharmaceuticals and the sweetener

As the urine was collected from a real system, the presence of pharmaceuticals and the sweetener sucralose before spiking was variable and is shown in Table 4 for the urine solutions collected in November 2021 and used in the PAC experiments, before and after spiking of the micropollutant mix.

**Table 4:** Initial concentration of the pharmaceuticals and the sweetener in the urine solutions used for the PAC experiment (collected in November 2021). Concentrations below limits of quantification are labeled as <LOQ. LOQs are given for urine after a 1:100 dilution.

	**Substance**	**LOQ in 100x diluted urine**	**Anaerobically stored urine**	**Organics-depleted urine**	**Anaerobically stored urine**	**Organics-depleted urine**
			**Before spiking**		**After spiking**	
		µg L^−1^	µg L^−1^	µg L^−1^	µg L^−1^	µg L^−1^
AMS	Amisulpride	0.2	<LOQ	6.6	168	161
ATA	Atenolol acid	0.6	193	33.8	410	243
ATE	Atenolol	0.2	<LOQ	<LOQ	166	161
CAN	Candesartan	0.7	3.8	2.8	171	158
CAR	Carbamazepine	0.1	<LOQ	0.2	176	166
CIT	Citalopram	0.1	2.4	4.9	152	155
CLA	Clarithromycin	0.3	<LOQ	<LOQ	162	153
DAR	Darunavir	2.1	<LOQ	<LOQ	167	162
DCF	Diclofenac	0.2	<LOQ	5.5	164	164
EMT	Emtricitabine	3.4	<LOQ	203.0	166	361
FEX	Fexofenadine	0.2	4.6	7.3	119	120
HCT	Hydrochlorothiazide	1.8	21.0	<LOQ	296	238
IRB	Irbesartan	0.2	<LOQ	<LOQ	163	145
LID	Lidocaine	0.1	<LOQ	2.2	162	161
MET	Metoprolol	0.1	15.4	2.6	186	162
NSMX	N_4_-Acetylsulfamethoxazole	0.5	<LOQ	<LOQ	173	167
SUC	Sucralose	11.4	<LOQ	64.1	211	271
SMX	Sulfamethoxazole	0.1	<LOQ	7.3	151	166
TMP	Trimethoprim	0.1	<LOQ	<LOQ	151	140
VEN	Venlafaxine	1.2	<LOQ	7.5	171	147
ATE&ATA	Atenolol & Atenolol Acid		193	33.8	577	404
NSMX&SMX	N_4_-Acetylsulfamethoxazole & Sulfamethoxazole		<LOQ	7.3	323	333

The selection of the substances included the substances required to be tested for advanced treatment performance by the Swiss water protection act (Bourgin et al., 2018; FOEN, 2015): amisulpride (AMS), candesartan (CAN), carbamazepine (CAR), citalopram (CIT), clarithromycin (CLA), diclofenac (DCF), hydrochlorothiazide (HCT), irbesartan (IRB), metoprolol (MET), and venlafaxine (VEN). The corrosion inhibitors benzotriazole and 4/5-methylbenzotriazole were not spiked, because they are not expected in urine. Furthermore, other substances were dosed, which are often found in urine (Bischel et al., 2015; Lienert et al., 2007; Özel Duygan et al., 2021): atenolol acid (ATA) & atenolol (ATE), darunavir (DAR), emtricitabine (EMT), fexofenadine (FEX), lidocaine (LID), N_4_-sulfamethoxazole (NSMX) & sulfamethoxazole (SMX), trimethoprim (TMP) and an artificial sweetener, sucralose (SUC).

A micropollutant mix with a concentration of 100 mg L^−1^ of each pharmaceutical and the sweetener was produced with available 1 g L^−1^ stock solutions of the individual substances. Most substances were dissolved in ethanol in their stock solution as they were not well soluble in water. To reduce the effect of the ethanol on the DOC, 90% of the ethanol in the mix was evaporated and replaced with nano-pure water (for DOC of ethanol, see chapter 4.2). A micropollutant concentration of 200 μg L^−1^ was chosen for spiking because it allows to still quantify a 95% removal for the substance with the highest LOQ. The expected concentrations in urine (as calculated from concentration in wastewater, see Table S5 in S9) range from 5 to 300 µg L^−1^ for the different substances.

### Analytical methods and calculations

For the measurement of the pharmaceuticals and the sweetener, a calibration was prepared in matrix water which ranged from 1 to 5000 ng L^−1^. The frozen samples were thawed, diluted 1:100 in nanopure water and spiked with a mixture of isotopically labeled internal standards (ISTD) at a final concentration of 200 ng L^−1^. Two samples were prepared in triplicates to determine the precision (3% on average). Liquid chromatography – triple quadrupole mass spectrometry (LC-MS/MS, Agilent TQ6495C) was used for analysis as described by Hagemann et al. (2020) with some modifications, explained in detail in section S6 in the SI together with the description of the quantification method, the evaluation of the relative recoveries, matrix effect and LOQs. Relative recoveries ranged from 78% to 132% for compounds with own ISTD and 93% to 117% for the three compounds without (see Table S3 in S6). The final concentrations of compounds without own ISTD were corrected by relative recovery. LOQs ranged from 0.8 to 114 ng L^−1^, with an average of 11 ng L^−1^ and a median of 2.7 ng L^−1^ over all measurements and without considering the 1:100 dilution of the urine samples.

Cations, anions and volatile fatty acids were measured with ion chromatography (881 compact IC pro, Metrohm) and DOC was measured with a total organic carbon analyzer (Shimadzu TOC-L). UV-absorbance was measured using a UV–VIS spectrophotometer (Agilent Cary 60) and the different fractions of the organics were characterized using size exclusion chromatography (SEC) (DOC-Labor GmbH) according to the method described by Huber et al. (2011). More details on the analytical methods can be found in section S7 in the SI.

Data analysis and calculations were done using MATLAB (MATLAB, 2020). Pharmaceutical concentrations, DOC, and UV absorbance were determined in the spiked samples before PAC addition and after the three days of the experiment. The measurements after three days in the batch without addition of PAC served as the reference for the PAC experiments. Changes within the three days without PAC addition were small and described in detail in section S3 in the SI. The removal (R) was calculated according to Eq. (1) with the concentration of a substance at a given PAC concentration (C) after three days and with the concentration in the reference batch without PAC addition (C_ref_) after three days.

(1) $$R=\left(1-\frac{C}{C_{ref}}\right)*100\;\left[\%\right]$$

Adsorption of pharmaceuticals on activated carbon can be described by isotherms, such as the Freundlich isotherm, which is most often used in water and wastewater treatment (Worch, 2021). However, the isotherm is for single solute adsorption. To include competition with other solutes a model is needed to describe multicomponent adsorption. The conventional approach to model multicomponent adsorption is the Ideal Adsorbed Solution Theory (IAST) that requires information about component composition, initial concentration, and the single-solute isotherm characteristics for each constituent. Since this information was not available for the urine solutions used in this study, the model could not be applied. To address this limitation, a modified technique called the Simplified Equivalent Background Compound Model (SEBCM) was used (Qi et al., 2007). The SEBCM is based on the Equivalent Background Compound Model (EBCM), which is a simplification of the IAST model. All organics are simplified into a solitary component termed the Equivalent Background Compound (EBC), which leads to the establishment of a competitive dual-component adsorption system involving the EBC and the pharmaceuticals. Consequently, only the isotherm parameters pertaining to single-solute adsorption and the initial concentrations of both components (EBC and pharmaceutical) are essential for describing the adsorption procedure. When the micropollutant concentration is significantly lower than that of natural organic matter (NOM), such as in urine, the EBCM can be further simplified to the SEBCM, because the adsorption capacity of NOM becomes dominant within the system (q_NOM_ >> q_MP_). A further assumption of the SEBCM is that the exponent n of the Freundlich isotherm of the organic matter and the pharmaceuticals are similar (n_NOM_ ≈ n_MP_), as presented and validated by Qi et al. (2007). The detailed derivation and the underlying assumption are well described in Worch (2021)

The fitting of the SEBCM parameters A and n was done using Eq. 2 (Worch, 2021), excluding the data for which less than 10% removal was observed in order to prevent numerical problems with calculated negative removal. To prevent division by zero (Eq. 2) the data points for 100% removal were also excluded.

(2) $$\ln\left(\frac{C_{ref}}{C}-1\right)=\frac{1}{n}\ln\left(\frac{m_{PAC}}{V_{urine}}\right)-\ln\left(A\right)\;$$

For representation, Eq. (2) was rearranged to show the removal as in Eq. (3).

(3) $$\left(1-\frac{C}{C_{ref}}\right)*100=\left(1-\frac{A}{{\left(\frac{m_{PAC}}{V_{urine}}\right)}^{1\;/\;n}+A}\;\right)*100\;\left[\%\right]$$

## Declaration of Competing Interest

Aurea Heusser and Kai Udert have submitted a patent, which includes separation of organics degradation and nitrification of urine. Furthermore, Kai Udert is co-owner of the company VunaNexus AG, which commercializes the Vuna process.

The authors declare neither the patent nor the involvement in the company VunaNexus have influenced the experiments, the evaluation or the interpretation of the work reported in this publication.

## Data Availability

The data are available in the digital appendix.

## Appendix Supplementary materials

Image, application 1

Image, application 2
