# Supplementary material for: High content of low molecular weight organics does not always affect pharmaceutical adsorption on activated carbon: The case of acetate, propionate and ethanol in source-separated urine
Source: Water Res X. 2023 Sep 7;21:100199. doi: 10.1016/j.wroa.2023.100199 (PMC10719575; doi:10.1016/j.wroa.2023.100199)
Supplement: Supplementary file 2 [file mmc2.docx]

**Supporting Information for**

**High content of low molecular weight organics does not always affect pharmaceutical adsorption on activated carbon: the case of acetate, propionate and ethanol in source-separated urine**

Aurea Heusser^a,b^, Anne Dax^a^, Christa S. McArdell^a^, Kai M. Udert^a,b^ *

^a^ Eawag, Swiss Federal Institute of Aquatic Science and Technology, 8600 Dübendorf, Switzerland

^b^ ETH Zürich, Institute of Environmental Engineering, 8093 Zürich, Switzerland

^*^ Corresponding author: Kai M. Udert, [Kai.Udert@eawag.ch](mailto:Kai.Udert@eawag.ch)

Submitted for publication in: Water Research X

List of contents

[S1 Size exclusion chromatography (SEC) 3](#_Toc138232182)

[S2 Adsorption of nutrients and other inorgancic compounds on PAC 4](#_Toc138232183)

[S3 Pharmaceutical removal without PAC 5](#_Toc138232184)

[S4 Removal analysis and corrections 6](#_Toc138232185)

[S5 Indicators 7](#_Toc138232186)

[S6 Pharmaceutical analytics 11](#_Toc138232187)

[S7 Analytics 12](#_Toc138232188)

[S8 Characteristics of the granular activated carbon 13](#_Toc138232189)

[S9 Expected concentrations of the pharmaceuticals and the sweetener in urine 13](#_Toc138232190)

[References 15](#_Toc138232191)

# Size exclusion chromatography (SEC)

The size exclusion chromatogram presented in Figure S 1B, shows anaerobically stored and organics-depleted urine spiked with the pharmaceutical mix. Due to the addition of ethanol a strong peak was detected in the neutral fraction (RT at 73 min). This peak was missing in all samples of anaerobically stored urine and organics-depleted urine without pharmaceutical spiking (Figure S 1A). Anaerobically stored urine showed a small peak around 88 minutes which might be attributed to creatinine or urea (Ruhl and Jekel, 2012) assuming that the hydrolysis was not completed during storage as those are substances known to be abundant in fresh urine (Udert et al., 2006).


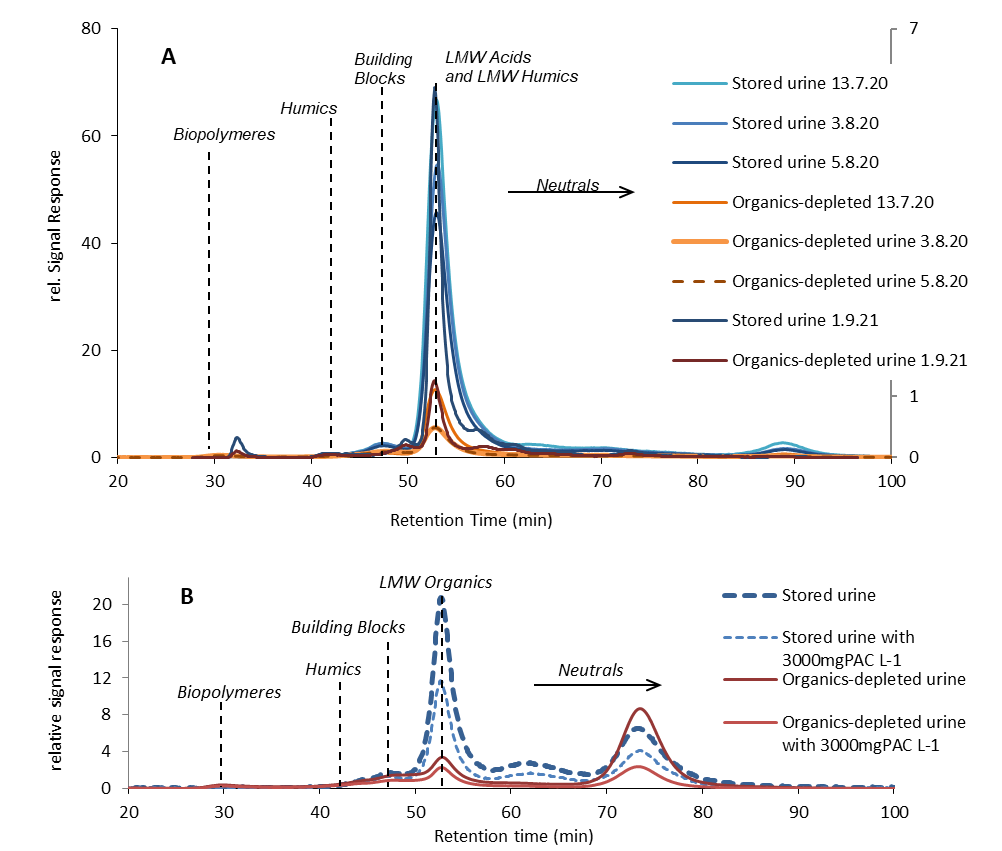


Figure S 1 A: Size exclusion chromatogram for anaerobically stored urine and organics-depleted urine sampled at different dates (samples from the 1. Sep 21 are depicted with the right y-axis and were measured by the DOC-Labor GmbH). B: Figure 4D from the main manuscript showing the size exclusion chromatogram for anaerobically stored urine and organics-depleted urine (collected in November 2021), spiked with pharmaceuticals in ethanol (peak at 73min) and treated with different concentrations of powdered activated carbon (PAC).

For organics-depleted urine, the effect of powdered activated carbon (PAC) addition (second set of PAC experiments collected in March 2021) is presented in Figure S 2. The organics-depleted urine before the experiment and after three days in the overhead shaker without PAC show a very similar chromatogram. Neither chemical nor biological processes resulted in a change of the organics fraction. As expected based on wastewater literature (Zietzschmann et al., 2014), the organics with smaller molecular weight adsorbed more to PAC and the biopolymers were hardly affected by the addition of PAC. Similar results were also found for studies on drinking water (Gibert et al., 2013) and lake water (Velten et al., 2011).


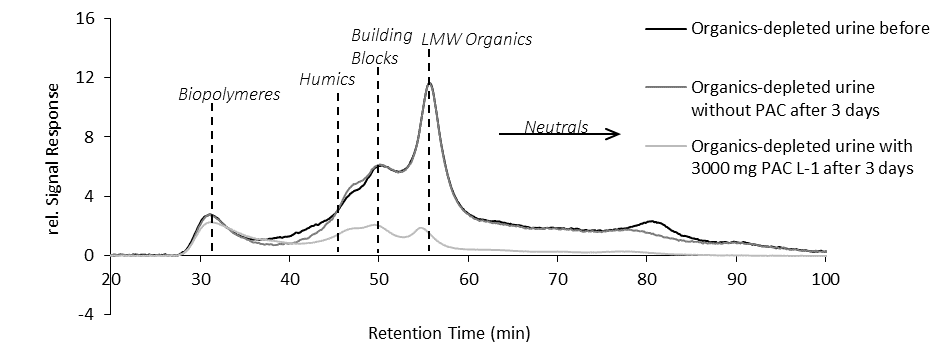


Figure S 2 Chromatograms of non-spiked organics-depleted urine before and after three days in the overhead shaker with and without PAC addition (urine collected March 2021).

# Adsorption of nutrients and other inorganic compounds on PAC

Concentrations of several inorganic compounds were measured in the different urine solutions with ion chromatography after filtration and dilution. Given the standard deviation of the measurements, no significant removal was observed (Figure S 3). For the nutrients NH_4_^+^ and PO_4_^3-^, no significant removal was detected neither, hence, the nutrients did not adsorb to activated carbon.

Figure S 3 Removal of inorganic compounds at different PAC concentrations from anaerobically stored urine, organics-depleted urine and organics-depleted urine with additional ethanol (collected in November 2021).

# Pharmaceutical removal without PAC

To calculate the removal of pharmaceuticals, the concentration in the batch after three days without PAC addition is used as a reference, to prevent interference by other processes than adsorption. The concentration of most pharmaceuticals remains the same (< 20% due to analytical uncertainties) over the duration of the experiment and without PAC addition as shown in Figure S 4. The only exceptions are citalopram (CIT), hydrochlorothiazide (HCT) and irbesartan (IRB).

Degradation of CIT is enhanced in alkaline conditions, but still slow with a half-life of 65 days (Kwon and Armbrust, 2005). No degradation was observed in anaerobically stored urine and organics-depleted urine, but 38% in organics-depleted urine with additional ethanol, despite similar high pH. Therefore, a measurement error might have occurred. For HCT, a half-live during abiotic storage of 20 days was found (Özel Duygan et al., 2021), what would explain a removal of up to 15% in 3 days. The 25% removal in anaerobically stored urine seems to be additionally influenced by analytical uncertainties. IRB was degraded by 30% to 45% in all samples. In literature, a half-life of decomposition at pH 9 of 21 hours at 25°C was reported (Mbah, 2004). The temperature in our 3-day experiments was lower, around 20°C, probably explaining our lower decomposition.

Figure S 4 Removal of pharmaceuticals, the sweetener and DOC after three days of the experiment without PAC in (1) anaerobically stored urine; (2) organics-depleted urine; (3) organics-depleted urine with additional ethanol. pH values in the solutions varied between 8.8 and 9. ATE & ATA is the sum of atenolol and atenolol acid and NSMX &SMX is the sum of sulfamethoxazole and N_4_-acetylsulfamethoxazole and "All pharmaceuticals" includes also the sweetener sucralose.

# Pharmaceutical adsorption and curve fitting

Darunavir (DAR) in anaerobically stored urine showed a constant removal of about 40% at PAC concentrations between 45 mg L^-1^ to 220 mg L^-1^ when referring to the reference concentration in the batch without PAC addition after three days (see Figure 2). In this reference sample, the same concentration as before the experiment was measured, *i.e.* no removal was observed over three days (without PAC addition). The high removal at low PAC doses, however, suggests a removal, which was not due to sorption, indicating a measurement error in the reference sample. The calculation of the removal using the DAR concentration at a PAC concentration of 45 mg L^-1^ as a reference (Figure S 5) led to the same removal as we observed in organics-depleted urine as shown in Figure S 6 and Table S 2. Apart from DAR also HCT and VEN showed a removal at low PAC concentrations not in anaerobically stored urine but in organics-depleted urine, also indicating an underestimation of the reference removal in organics-depleted urine without PAC (Figure S 5). The correction of HCT and VEN removal by referring to the HCT and VEN concentration in the sample with 45 mg PAC L^-1^ as shown in Figure S 6 led to an overlap of removal in the two urine solutions (Table S 2). Candesartan (CAN) is the substance tested with the highest carbon requirement for adsorption. At a PAC concentration of 1300 mg L^-1^ we observed only 10% removal for both, anaerobically stored and organics-depleted urine. At the higher PAC concentrations of 2000 mg L^-1^ and 3000 mg L^-1^ the removal from anaerobically stored urine was better compared to organics-depleted urine. However, more data points at higher PAC concentrations would be needed to evaluate the difference between the two urine solutions.

Figure S 5 Concentration of darunavir in stored urine and hydrochlorothiazide and venlafaxine in organics-depleted urine at different PAC concentrations after three days. The concentration without PAC addition is depicted in a red circle and considered to be an outlier. Instead, the concentration indicated with a green circle was taken as reference sample.

CIT had a better removal without the presence of additional ethanol. However, as shown in Figure S 4 we observed a removal of almost 40% without PAC addition for the organics-depleted urine with ethanol, but none for anaerobically stored and organics-depleted urine. If we adapt the reference to the measurement before the three days of experiment, the removal with and without ethanol was the same as shown in Figure S 6.

Figure S 6 Removal of darunavir with corrected reference (45mgPAC L^-1^ instead of 0 mg PAC L^-1^) for anaerobically stored urine, removal of hydrochlorothiazide and venlafaxine with corrected reference (45mgPAC L^-1^ instead of 0 mg PAC L^-1^) for organics-depleted urine and citalopram with corrected reference (before the experiment instead of 0 mg PAC L^-1^) for organics-depleted urine with ethanol. See text for details.

After completion of the data correction the curve fitting was done using the SEBCM with Equation 2 as shown in Figure S 7 and Figure S 8. The goodness of fit and the estimated parameters are shown in Table S 1 and the test of the confidence intervals of the estimated parameters overlap is shown in Table S 2. Table S 2 reveals that only the slope of the linear regression fit for clarithromycin does not overlap. Hence, for almost all substances the two fitted curves lie within their 95% confidence interval.

Figure S 7 Fitting of removal data according to SEBCM, the markers without filling represent data with removal below 10% and were not included in the fitting, the data where the removal is 100% is not depicted. Substances marked with an asterisk (*) have the batch with 45 mg PAC as a reference (see Figure S 5).

Figure S 8 Fitting of removal data according to SEBCM for organics-depleted urine, the markers without filling represent data with removal below 10% and were not included in the fitting, the data where the removal is 100% is not depicted. Substances marked with an asterisk (*) have the batch with 45 mg PAC as a reference (see Figure S 5). Organics-depleted urine with ethanol has not enough data points for a curve fitting.

Table S 1 Goodness of fit and fitting parameters for the linear regression of the pharmaceuticals in organics-depleted urine and stored urine. Including the number of observations (Num. Obs.), the R-squared (R^2^) and the root mean square error (RMSE).

|  | **Organics-depleted urine** | | | | | **Anaerobically stored urine** | | | | |
| --- | --- | --- | --- | --- | --- | --- | --- | --- | --- | --- |
| **Parameter** | Num Obs | R^2^ | RMSE | log(A) | n^-1^ | Num Obs | R^2^ | RMSE | log(A) | n^-1^ |
| **Amisulpride** | 11 | 0.87 | 0.45 | -6.42 | 2.76 | 8 | 0.98 | 0.19 | -9.87 | 4.20 |
| **Atenolol acid** | 7 | 0.91 | 0.39 | -9.57 | 3.49 | 6 | 0.98 | 0.20 | -13.73 | 4.89 |
| **Atenolol** | 11 | 0.96 | 0.18 | -3.70 | 1.92 | 10 | 0.98 | 0.16 | -5.68 | 2.80 |
| **Candesartan** | 1 | NaN | 0.00 | 0.00 | -0.08 | 2 | 1.00 | 0.00 | -18.03 | 5.51 |
| **Carbamazepine** | 13 | 0.92 | 0.36 | -5.30 | 2.28 | 10 | 0.97 | 0.21 | -7.89 | 3.38 |
| **Citalopram** | 15 | 0.87 | 0.37 | -3.22 | 1.57 | 15 | 0.89 | 0.50 | -4.55 | 2.24 |
| **Clarithromycin** | 4 | 0.76 | 0.27 | -5.56 | 1.70 | 6 | 0.99 | 0.13 | -9.03 | 3.18 |
| **Darunavir** | 5 | 0.94 | 0.27 | -12.35 | 4.48 | 12 | 0.75 | 0.27 | -2.17 | 0.96 |
| **Diclofenac** | 7 | 0.89 | 0.42 | -9.44 | 3.36 | 6 | 0.98 | 0.23 | -13.19 | 4.74 |
| **Emtricitabine** | 10 | 0.94 | 0.27 | -6.27 | 2.52 | 8 | 0.98 | 0.11 | -5.82 | 2.31 |
| **Fexofenadine** | 5 | 0.82 | 0.52 | -10.13 | 3.57 | 6 | 0.88 | 0.44 | -11.74 | 4.28 |
| **Hydrochlorothiazide** | 12 | 0.81 | 0.37 | -3.53 | 1.59 | 7 | 0.96 | 0.15 | -6.55 | 2.60 |
| **Irbesartan** | 4 | 0.88 | 0.30 | -8.86 | 2.94 | 4 | 0.98 | 0.25 | -18.55 | 6.24 |
| **Lidocaine** | 12 | 0.89 | 0.42 | -5.54 | 2.37 | 8 | 0.96 | 0.24 | -8.56 | 3.59 |
| **Metoprolol** | 15 | 0.91 | 0.30 | -2.59 | 1.57 | 12 | 0.98 | 0.20 | -5.25 | 2.81 |
| **N_4_-acetylsulfamethoxazole** | 4 | 0.88 | 0.30 | -8.56 | 2.87 | 4 | 1.00 | 0.05 | -17.35 | 5.62 |
| **Sucralose** | 2 | 1.00 | 0.00 | -20.48 | 7.04 | 2 | 1.00 | 0.00 | -23.17 | 7.69 |
| **Sulfamethoxazole** | 5 | 0.92 | 0.25 | -8.28 | 2.70 | 3 | 1.00 | 0.03 | -14.08 | 4.43 |
| **Trimethoprim** | 14 | 0.91 | 0.42 | -5.13 | 2.31 | 8 | 0.97 | 0.19 | -8.78 | 3.74 |
| **Venlafaxine** | 9 | 0.74 | 0.42 | -5.37 | 2.12 | 5 | 0.98 | 0.10 | -9.71 | 3.71 |
| **ATE & ATA** | 14 | 0.87 | 0.43 | -4.57 | 1.93 | 10 | 0.88 | 0.41 | -6.82 | 2.67 |
| **NSMX & SMX** | 4 | 0.87 | 0.27 | -7.57 | 2.53 | 3 | 1.00 | 0.01 | -14.12 | 4.52 |
| **All Pharmaceuticals** | 11 | 0.93 | 0.17 | -3.75 | 1.38 | 11 | 0.99 | 0.07 | -4.16 | 1.56 |

Table S 2 Analysis if the 95% confidence intervals of the estimated parameters, slope and intercept of the linear regressions for anaerobically stored and organics-depleted urine presented in Figure S 7, overlap for all substances.

|  | **Overlap of confidence interval** | |
| --- | --- | --- |
|  | **Slope** | **Intercept** |
| **Amisulpride** | yes | yes |
| **Atenolol acid** | yes | yes |
| **Atenolol** | yes | yes |
| **Candesartan** | - | - |
| **Carbamazepine** | yes | yes |
| **Citalopram** | yes | yes |
| **Clarithromycin** | no | yes |
| **Darunavir** | yes | yes |
| **Diclofenac** | yes | yes |
| **Emtricitabine** | yes | yes |
| **Fexofenadine** | yes | yes |
| **Hydrochlorothiazide** | yes | yes |
| **Irbesartan** | yes | yes |
| **Lidocaine** | yes | yes |
| **Metoprolol** | yes | yes |
| **N_4_-acetylsulfamethoxazole** | yes | yes |
| **Sucralose** | yes | yes |
| **Sulfamethoxazole** | yes | yes |
| **Trimethoprim** | yes | yes |
| **Venlafaxine** | yes | yes |
| **ATE & ATA** | yes | yes |
| **NSMX & SMX** | yes | yes |
| **All Pharmaceuticals** | yes | yes |

# Indicators

Measurements of acetate, propionate, ethanol and the pharmaceutical mix, each dissolved in nanopure water, demonstrated that only pharmaceuticals but neither acetate, propionate nor ethanol absorb UV light substantially at 265 nm and 254 nm, highlighted with the black diamonds in Figure S 9.


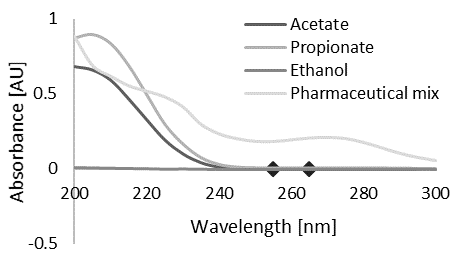


Figure S 9 UV absorbance of acetate, propionate, ethanol and the pharmaceutical mix, each in nanopure water. The black diamonds highlight the wavelength of 245 nm and 265 nm.

Figure S 10 and Figure S 11 show the removal of pharmaceuticals as function of UV_265_ absorption and the dissolved organic carbon (DOC) removal, respectively.

Figure S 10 Correlation of the individual pharmaceutical removal and UV_265_ absorbance removal and the correlation of DOC removal and UV_265_ absorbance removal. Measurements in anaerobically stored and organics-depleted urine with different PAC additions between 0-3000 mg L^-1^ (collected July and March 2021). ATE & ATA is the sum of atenolol and atenolol acid and NSMX &SMX is the sum of sulfamethoxazole and N_4_-acetylsulfamethoxazole and "All pharmaceuticals" includes also the sweetener sucralose.

Figure S 11 Correlation of the individual pharmaceutical removal and DOC removal and the correlation of UV_265_ absorbance removal and DOC removal. Measurements in anaerobically stored and organics-depleted urine with different PAC additions between 0-3000 mg L^-1^ (collected July and March 2021). ATE & ATA is the sum of atenolol and atenolol acid and NSMX &SMX is the sum of sulfamethoxazole and N_4_-acetylsulfamethoxazole and "All pharmaceuticals" includes also the sweetener sucralose.

**Materials and methods**

# Pharmaceutical analytics

For the measurement of the pharmaceuticals, a calibration, which ranged from 1 to 5000 ng L^-1^, was prepared in matrix water. The matrix water was prepared by adding a set amount of salts and urea in nanopure water (see Lee et al. (2021) for details). The resulting matrix water mirrors the tap water in Dübendorf without contamination.

The frozen samples were thawed, diluted 1:100 in nanopure water and spiked with a mixture of isotopically labeled internal standards (ISTD) at a final concentration of 200 ng L^-1^, before being shaken and vortexed to assure mixing. 8 mL were transferred to centrifuge vials and centrifuged for 15 minutes at 4000 rpm. The supernatant was then transferred to LC vials. Two samples were prepared in triplicates to evaluate precision (reproducibility of the sample preparation as well as the robustness of the analysis). Relative standard deviations of the triplicates ranged from 0.3 to 12% (which was one outlier), with an average of 3% and a median of 2%.

To assess relative recovery of the analytes, randomly selected samples were spiked to contain 1 µg/L of the standard solution.

Liquid chromatography – triple quadrupole mass spectrometry (LC-MS/MS) was used for analysis as described by Hagemann et al. (2020) with some modifications. The samples were measured by direct injection of 100 µL onto an Agilent 1290 Infinity LC system. Chromatographic separation was performed on a Acquity UPLC HSS T3 column (1.8 µm, 3.0x100 nm, Waters) and detection on a triple quadrupole MS (Agilent TQ6495C and Agilent TQ6495A). The electrospray ionization was operated with a capillary voltage of 3500 V in positive and 3000 V in negative mode and a dynamic MRM with 650 ms cycle time. The LC System was operated at a flow rate of 0.5 mL min^-1^ with a gradient of 100% eluent A (nanopure water plus 0.1% formic acid) to 95% eluent B (methanol plus 0.1% formic acid) in 18.5 min, hold for 3.5 min, and then go to 100% eluent A in 0.5 min and hold for 4.5 min.

The quantification of the measured compounds was performed using the software MassHunter Quantitative Version 10.1 for QQQ (Agilent Technologies). Two transitions were used for quality control and the qualifier recovery was calculated as the ratio between quantifier and qualifier transition, using a tolerance level between 80% and 120%.

The data was measured in three sessions, in April and August 2021 and January 2022. The LOQs are shown as a range of these sessions, the relative recoveries as mean value with standard deviation in Table S1. .

Relative recoveries ranged from 78% to 132% for compounds with own ISTD and 93% to 117% for compounds without (Table S 2). The final concentrations of compounds without own ISTD were corrected by relative recovery. For Metoprolol and Sucralose, no matching internal standard was available, for Darunavir, a matching one was available, but was unstable in the measurements. Internal standards for these three substances were chosen from those available based on similar retention time and best relative recovery.

Matrix factors for compounds with own ISTD were generated by dividing the area of the ISTD in the sample by the average of the areas of the ISTDs in the calibration. For compounds without their own ISTD, the area of an unspiked sample was subtracted from the area of the corresponding spiked sample. The result was then divided by the average of the areas of the calibration points with the same concentration.

For calculating the limit of quantification (LOQ), the lowest found calibration standard was divided by the matrix factor. LOQs ranged from 0.8 to 114 ng L^-1^, with an average of 11.1 ng L^-1^ and a median of 2.7 ng L^-1^ (Table S 2). Samples were measured with a 1:100 dilution, and the concentrations of the relevant LOQs were later calculated back. Due to this dilution factor, concentrations of compounds might have slipped below the detection limit.

Table S 3 Limits of quantification (LOQ in ng L^-1^), relative recovery (in %) and information on isotopically labelled internal standard for analysed pharmaceuticals. LOQ and relative recovery are given in ranges to reflect the three sessions in which the samples were measured.

|  | **LOQ** | **Rel. Recovery** | **Own ISTD** | **ISTD** |
| --- | --- | --- | --- | --- |
| Amisulpride | 2 – 3 | 115 ± 12 | Yes | Amisulpride-D5 |
| Atenolol acid | 2 – 6 | 94 ± 10 | Yes | Atenolol acid-D5 |
| Atenolol | 2 – 3 | 115 ± 12 | Yes | Atenolol-D7 |
| Candesartan | 5 – 7 | 94 ± 12 | Yes | Candesartan-D5 |
| Carbamazepine | 1 – 5 | 101 ± 4 | Yes | Carbamazepin-D8 |
| Citalopram | 1 – 3 | 106 ± 8 | Yes | Citalopram-D6 |
| Clarithromycin | 1 – 3 | 118 ± 10 | Yes | Clarithromycin-D3 |
| Darunavir | 5 – 21 | 94 ± 9 | No | Fexofenadine-D6 |
| Diclofenac | 2 – 5 | 98 ± 8 | Yes | Diclofenac-D4 |
| Emtricitabine | 34 – 57 | 99 ± 11 | Yes | Emtricitabine-13C15N2 |
| Fexofenadine | 1 – 2 | 93 ± 15 | Yes | Fexofenadine-D6 |
| Hydrochlorthiazid | 9 – 18 | 96 ± 10 | Yes | Hydrochlorthiazid-13C,D2 |
| Irbesartan | 1 – 2 | 99 ± 6 | Yes | Irbesartan-D4 |
| Lidocaine | 1 – 2 | 99 ± 12 | Yes | Lidocain-D10 |
| Metoprolol | 1 – 2 | 102 ± 7 | No | N4-Acetylsulfamethoxazol-D4 |
| N4-acetylsulfamethoxazole | 5 | 95 ± 9 | Yes | N4-Acetylsulfamethoxazol-D4 |
| Sucralose | 78 – 114 | 102 ± 11 | No | Sulfamethoxazol-D4 |
| Sulfamethoxazole | 1 – 2 | 100 ± 7 | Yes | Sulfamethoxazol-D4 |
| Trimethoprim | 1 – 2 | 101 ± 7 | Yes | Trimethoprim-D3 |
| Venlafaxine | 12 – 21 | 102 ± 17 | Yes | Venlafaxin-D6 |

# Analytics

The cations sodium, ammonium and potassium and the anions chloride, nitrate and phosphate were measured with ion chromatography (881 compact IC pro, Metrohm) after a 1:100 dilution. DOC was measured with a total organic carbon analyzer (Shimadzu TOC-L) with 1:500 dilution for anaerobically stored urine and 1:100 for organics-depleted urine. For the samples treated with PAC, the dilutions were adapted to meet a range of measurement of 0.5-10 mg C L^-1^. UV-absorbance was measured using a UV-VIS spectrophotometer (Agilent Cary 60, Agilent Technologies) after a 1:10 dilution for all samples. The device measured the range of 200-800 nm but only the data at 265 nm are presented, which is the wavelength preferred to prevent the influence of nitrate on the measurement (Köpping et al., 2020). For each series of measurements, a blank with nanopure was measured as well and the sample measurement results were corrected by subtracting the blank measurement. The different fractions of the organics were characterized using size exclusion chromatography (SEC) – organic carbon detection (OCD) – organic nitrogen detection (OND) according to the method described by Huber et al. (2011). The dilutions varied depending on the measured DOC between 1:1000 and 1:25. The DOC, as well as the organics fractions were identified and quantified using FIFFIKUS (DOC-Labor Dr. Huber). The organics acetate, propionate, iso-butyrate, butyrate, iso-valerate, and valerate were quantified with ion chromatography (881 compact IC pro, Metrohm) after a 1:50 dilution for anaerobically stored urine and 1:20 for organics-depleted urine with a limit of quantification of 2 ppm of each substance. Ethanol was not quantified, but the DOC increase by the addition of ethanol was measured

# Characteristics of the granular activated carbon

The presented characteristics (Table S 3) are for the activated carbon in granular form, for our experiment the granular activated carbon (GAC) was milled to get PAC.

Table S 4 Characteristics of the granular activated carbon Norit® GCN 830.

| Iodine Number | - | 1050 |
| --- | --- | --- |
| Particle size > 2.36 mm | mass-% | max. 5 |
| Particle size < 0.6 mm | mass-% | max. 5 |
| Moisture (as packed) | mass-% | max. 5 |
| Total surface area (B.E.T.) | m^2^ g^-1^ | 1150 |
| Apparent density | kg m^-3^ | 510 |
| Density backwashed and drained | kg m^-3^ | 450 |
| Ball-pan hardness | - | 99 |
| Effective size D_10_ | mm | 0.9 |
| Uniformity coefficient | - | 1.7 |
| Ash content | mass-% | 3 |
| pH | - | alkaline |
| Dechlorination halving value | cm | 5 |
| Point of zero charge* | pH_pzc_ | 8.6 |

*No producer information but from Racytè (2013)

# Expected concentrations of the pharmaceuticals and the sweetener in urine

To calculate the expected concentration of micropollutants in urine (C_urine_), the well known concentrations of the micropollutants in wastewater (C_wastewater_) were used. A flow (Q) of 350L p^-1^ d^-1^ for wastewater (Gujer, 2007) and 1.25 L p^-1^ d^-1^ for urine (Udert et al., 2006) was assumed. The fractions excreted from the human body in urine (F_urine_) and feces (F_feces_) were taken from literature (see Table S3). As these fraction not always sum up to 100%, the fraction in urine from wastewater (F) is corrected to then calculate the concentration expected in urine as shown in Table S 4.

$$F=\frac{F_{Urine}}{F_{Urine}+F_{Faeces}} , with F \left[ \% \right] being the fraction of micropollutant in urine from wastewater$$

$$C_{urine}=C_{wastewater}*F*\frac{Q_{wastewater}}{Q_{Urine}}$$

Table S 5 Concentration of the selected pharmaceuticals and the sweetener expected in urine. Expected concentrations were calculated from concentrations in wastewater (literature: a,b,c,d), wastewater and urine flow and the excretion ratios (literature: e,f,g,h).

| Substance | | Conc. in wastewater | | Excretion in Urine | | Faeces | | Urine + Faeces | | Fraction in urine from WW | | Conc. expected in urine* | | Literature | |
| --- | --- | --- | --- | --- | --- | --- | --- | --- | --- | --- | --- | --- | --- | --- | --- |
|  | | ug/L | | % | | % | | % | | % | | ug/L | | source | |
| Amisulpride | | 0.129 | | 26% | | 51% | | 77% | | 34% | | 12.2 | | c,f | |
| Atenolol | | 0.314 | | 37% | | 46% | | 83% | | 45% | | 39.2 | | a,b | |
| Atenolol acid | | 1.040 | | 100% | | 0% | | 100% | | 100% | | 291.0 | | a,b | |
| Candesartan | | 0.401 | | 26% | | 56% | | 82% | | 32% | | 35.6 | | a,b | |
| Carbamazepine | | 0.584 | | 2% | | 24% | | 26% | | 8% | | 12.6 | | a,b | |
| Citalopram | | 0.113 | | 19% | | 78% | | 97% | | 20% | | 6.2 | | c,f | |
| Clarithromycin | | 0.109 | | 25% | | 8% | | 33% | | 76% | | 23.1 | | a,b | |
| Diclofenac | | 0.971 | | 50% | | 0% | | 50% | | 100% | | 272.0 | | a,b | |
| Emtricitabine | | 0.280 | | 75% | | 14% | | 89% | | 84% | | 66.1 | | a,b | |
| Fexofenadine | | 0.189 | | 12% | | 80% | | 92% | | 13% | | 6.9 | | c,e | |
| Hydrochlorothiazide | | 1.250 | | 82% | | 18% | | 100% | | 82% | | 286.0 | | a,b | |
| Irbesartan | | 0.666 | | 1% | | 10% | | 11% | | 9% | | 17.0 | | a,b | |
| Lidocaine | | 0.195 | | 5% | | 0% | | 5% | | 100% | | 54.6 | | c, g | |
| Metoprolol | | 0.353 | | 7% | | 4% | | 11% | | 64% | | 62.9 | | a,b | |
| N_4_-acetyl-sulfamethoxazole | | 0.075 | | 50% | | 0% | | 50% | | 100% | | 21.0 | | a,b | |
| Sucralose | | 4.520 | | 8% | | 73% | | 81% | | 10% | | 125.0 | | c, h | |
| Sulfamethoxazole | | 0.235 | | 20% | | 0% | | 20% | | 100% | | 65.8 | | a,b | |
| Venlafaxine | | 0.195 | | 46% | | 0% | | 46% | | 100% | | 54.6 | | c,f | |
| Darunavir | | 0.495 | | 8% | | 41% | | 49% | | 16% | | 21.9 | | c,f | |
| Trimethoprim | | 0.045 | | 60% | | n.a. | | 60% | | 100% | | 12.6 | | a,b | |
| * Assuming 350L WW p^-1^ d^-1^ (Gujer, 2007) and 1.25 L Urine p^-1^ d^-1^ (Udert et al., 2006); a:(Otto et al., 2014) ; b: (Köpping et al., 2020); c: (Bourgin et al., 2018); d:(Bischel et al., 2015) ; e: (Aventis Pharmaceuticals Inc., 2007); f: (Eberhard et al., 2023, in preparation); g:(Collinsworth et al., 1974) h:(Roberts et al., 2000) | | | | | | | | | | | | | | | |

# References

Aventis Pharmaceuticals Inc. 2007 Prescribing Information ALLEGRA®(fexofenadine hydrochloride) Capsules and Tablets. Inc., A.P. (ed), Kansas City, MO 64137 USA.

Bischel, H.N., Duygan, B.D.Ö., Strande, L., McArdell, C.S., Udert, K.M. and Kohn, T. 2015. Pathogens and pharmaceuticals in source-separated urine in eThekwini, South Africa. Water Research 85, 57-65.

Bourgin, M., Beck, B., Boehler, M., Borowska, E., Fleiner, J., Salhi, E., Teichler, R., von Gunten, U., Siegrist, H. and McArdell, C.S. 2018. Evaluation of a full-scale wastewater treatment plant upgraded with ozonation and biological post-treatments: Abatement of micropollutants, formation of transformation products and oxidation by-products. Water Research 129, 486-498.

Collinsworth, K.A., Kalman, S.M. and Harrison, D.C. 1974. The clinical pharmacology of lidocaine as an antiarrhythymic drug. Circulation 50(6), 1217-1230.

Eberhard, Y., Maccagnan, A., Berg, A. and McArdell, C.S. 2023, in preparation. Antibiotics and other micropollutants in Swiss sewage sludge and fecal compost.

Gibert, O., Lefèvre, B., Fernández, M., Bernat, X., Paraira, M. and Pons, M. 2013. Fractionation and removal of dissolved organic carbon in a full-scale granular activated carbon filter used for drinking water production. Water Research 47(8), 2821-2829.

Gujer, W. (2007) Siedlungswasserwirtschaft (Urban Water Management), Springer, Berlin, Heidelberg.

Hagemann, N., Schmidt, H.-P., Kägi, R., Böhler, M., Sigmund, G., Maccagnan, A., McArdell, C.S. and Bucheli, T.D. 2020. Wood-based activated biochar to eliminate organic micropollutants from biologically treated wastewater. Science of the Total Environment 730, 138417.

Huber, S.A., Balz, A., Abert, M. and Pronk, W. 2011. Characterisation of aquatic humic and non-humic matter with size-exclusion chromatography–organic carbon detection–organic nitrogen detection (LC-OCD-OND). Water Research 45(2), 879-885.

Köpping, I., McArdell, C.S., Borowska, E., Böhler, M.A. and Udert, K.M. 2020. Removal of pharmaceuticals from nitrified urine by adsorption on granular activated carbon. Water Research X 9, 100057.

Kwon, J.W. and Armbrust, K.L. 2005. Degradation of citalopram by simulated sunlight. Environmental Toxicology and Chemistry: An International Journal 24(7), 1618-1623.

Lee, J., Ju, F., Maile-Moskowitz, A., Beck, K., Maccagnan, A., McArdell, C.S., Dal Molin, M., Fenicia, F., Vikesland, P.J. and Pruden, A. 2021. Unraveling the riverine antibiotic resistome: the downstream fate of anthropogenic inputs. Water Research 197, 117050.

Mbah, C. 2004. Kinetics of decomposition of irbesartan in aqueous solutions determined by high performance liquid chromatography. Die Pharmazie-An International Journal of Pharmaceutical Sciences 59(12), 920-922.

Otto, J., Singer, H., Vogler, B., Deuber, F., Longrée, P., Czekalski, N., McArdell, C. and Götz, C. 2014. Substanzen zur Überprüfung des Reinigungseffekts weitergehender Abwasserbehandlungsverfahren (Substances for testing the purification effect of more advanced wastewater treatment processes). Fachbericht im Auftrag des Bundesamts für Umwelt BAFU, Report, Eawag.

Özel Duygan, B.D., Udert, K.M., Remmele, A. and McArdell, C.S. 2021. Removal of pharmaceuticals from human urine during storage, aerobic biological treatment, and activated carbon adsorption to produce a safe fertilizer. Resources, Conservation and Recycling 166, 105341.

Racytè, J. (2013) Alternating field activated carbon fluidized bed electrode for water disinfection, Wageningen University and Research.

Roberts, A., Renwick, A., Sims, J. and Snodin, D. 2000. Sucralose metabolism and pharmacokinetics in man. Food and Chemical Toxicology 38, 31-41.

Ruhl, A.S. and Jekel, M. 2012. Elution behaviour of low molecular weight compounds in size exclusion chromatography. Journal of Water Supply: Research and Technology—AQUA 61(1), 32-40.

Udert, K., Larsen, T.A. and Gujer, W. 2006. Fate of major compounds in source-separated urine. Water Science and Technology 54(11-12), 413-420.

Velten, S., Knappe, D.R., Traber, J., Kaiser, H.-P., Von Gunten, U., Boller, M. and Meylan, S. 2011. Characterization of natural organic matter adsorption in granular activated carbon adsorbers. Water Research 45(13), 3951-3959.

Zietzschmann, F., Worch, E., Altmann, J., Ruhl, A.S., Sperlich, A., Meinel, F. and Jekel, M. 2014. Impact of EfOM size on competition in activated carbon adsorption of organic micro-pollutants from treated wastewater. Water Research 65, 297-306.
